# CHTOP in Chemoresistant Epithelial Ovarian Cancer: A Novel and Potential Therapeutic Target

**DOI:** 10.3389/fonc.2019.00557

**Published:** 2019-06-27

**Authors:** Xiaojie Feng, Xupeng Bai, Jie Ni, Valerie C. Wasinger, Julia Beretov, Ying Zhu, Peter Graham, Yong Li

**Affiliations:** ^1^Department of Gynaecological Oncology, Henan Cancer Hospital, Zhengzhou, China; ^2^Cancer Care Centre, St. George Hospital, Kogarah, NSW, Australia; ^3^St. George and Sutherland Clinical School, University of New South Wales Sydney, Sydney, NSW, Australia; ^4^Bioanalytical Mass Spectrometry Facility, Mark Wainwright Analytical Centre, University of New South Wales Sydney, Sydney, NSW, Australia; ^5^School of Medical Science, University of New South Wales Sydney, Sydney, NSW, Australia; ^6^Anatomical Pathology, NSW Health Pathology, St. George Hospital, Kogarah, NSW, Australia; ^7^School of Basic Medical Sciences, Zhengzhou University, Zhengzhou, China

**Keywords:** ovarian cancer, chemoresistance, CHTOP, apoptosis, stemness, metastasis

## Abstract

**Objective:** Chemoresistance is a major challenge in epithelial ovarian cancer (EOC) treatment. Chromatin target of protein arginine methyltransferase (CHTOP) was identified as a potential biomarker in chemoresistant EOC cell lines using label-free LC-MS/MS quantitative proteomics. Thus, the aim of this study is to investigate the role of CHTOP in chemoresistant EOC and the underlying mechanism.

**Methods:** The expression of CHTOP in human ovarian cancer cells and tissues was detected using immunofluorescence (IF), western blot (WB), and immunohistochemistry (IHC), respectively. Flow cytometry and TUNEL assay were employed to detect the effect of CHTOP knockdown (KD) in chemoresistant EOC cell apoptosis, while colony and sphere formation assays were used to evaluate its effect on cell stemness. The association of CHTOP with cell metastasis was determined using Matrigel invasion and wound-healing assays.

**Results:** The higher level expression of CHTOP protein was found in chemoresistant EOC cells as compared to their sensitive parental cells or normal epithelial ovarian cells. Results from IHC and bioinformatic analysis showed CHTOP was highly expressed in human ovarian cancer tissues and associated with a poor progression-free survival in patients. In addition, CHTOP KD significantly enhanced cisplatin-induced apoptosis, reduced the stemness of chemoresistant EOC cells, and decreased their metastatic potential.

**Conclusion:** Our findings suggest that CHTOP is associated with apoptosis, stemness, and metastasis in chemoresistant EOC cells and might be a promising target to overcome chemoresistance in EOC treatment.

## Introduction

Ovarian cancer is the most lethal gynecological malignancies worldwide. In 2017, over 22,000 women were diagnosed with ovarian cancer and ~ 14,080 died of the disease (1). Epithelial ovarian cancer (EOC) accounts for ~85–95% of all ovarian cancer cases, and more than 70% of EOC patients were diagnosed in the advanced stages due to poor early screening techniques (2).

Chemotherapy is the mainstay for the treatment of ovarian cancer. However, despite an initial high response rate to chemotherapy, over 85% of advanced EOC patients experienced cancer relapse within 18 to 24 months because of the clonal expansion of either acquired or innate drug-resistant tumor cells. The 5-year overall survival rate for advanced ovarian cancer is only ~ 30% (3). Therefore, chemoresistance is a major obstacle to long-term remission and effective strategies to overcome chemoresistance would have a positive clinical impact.

**Figure 1:**
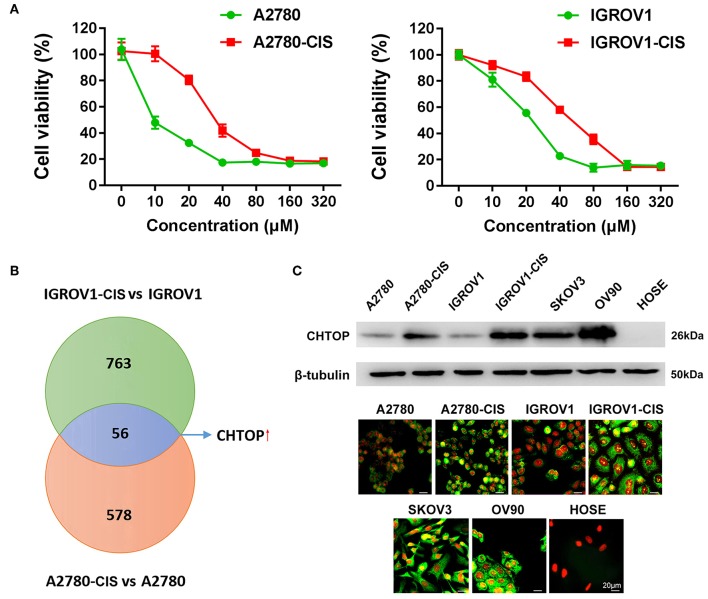
Proteomics identified CHTOP as a highly expressed protein in cisplatin-resistant EOC cells. **(A)** Cell viability was detected using MTT assay after treatment with various concentrations of cisplatin for 48 h. **(B)** The higher expression of CHTOP in cisplatin-resistant EOC cell lines was identified using label-free LC-MS/MS-based proteomics. **(C)** Higher protein expression of CHTOP was found in cisplatin-resistant EOC cell lines and metastatic EOC cell lines (SKOV3 and OV90). Negative expression of CHTOP was observed in normal epithelial ovary cell line (HOSE). CHTOP expression was detected by IF and WB. β-tubulin was used as the loading control. IF images were photographed at 400 × magnification. Green fluorescence represents CHTOP, while red fluorescence represents nucleus. Data were expressed as mean ± SD (*n* = 3).

Preliminary data from our label-free LC-MS/MS quantitative proteomics showed that chromatin target of protein arginine methyltransferase (CHTOP) was among the most up-regulated proteins in chemoresistant EOC cell lines (Figure 1B). CHTOP is a vertebrate-specific chromatin-bound protein that plays an important role in transcriptional regulation. It was found to be involved in the transcriptional activation of estrogen receptor-targeted genes such as *TFF1 (pS2)* in breast cancer cells and downregulation of fetal γ-globin during the developmental transition from fetal to adult hemoglobin (4, 5). Furthermore, CHTOP also acts as a component of TRanscription-EXport complex to process nascent pre-mRNA splicing and control mature mRNA export (5, 6). A recent study reported that CHTOP was recruited to 5-hydroxymethylcytosine-containing DNA sequences and involved in the tumorigenicity of glioblastoma (7), which suggests that CHTOP may be a potential therapeutic target for cancer therapy. However, the role of CHTOP in EOC remains unknown.

In this study, we aimed to investigate whether CHTOP can be used as a therapeutic target in chemoresistant EOC cells and to reveal the underlying mechanism. Our results showed that highly expressed CHTOP in ovarian cancer tissues was associated with an advanced stage and poor progression-free survival (PFS) in patients. Further results indicated that the overexpression of CHTOP was associated with apoptosis, stemness, and metastasis in chemoresistant EOC cells. These findings suggest that CHTOP might be a promising therapeutic target for overcoming EOC chemoresistance.

## Materials and Methods

### Cell Culture

EOC cell lines (A2780, IGROV1, SKOV-3, and OV-90) were obtained from American Type Culture Collection (ATCC, Rockville, MD, USA). Cisplatin-resistant EOC cell line A2780-cis was purchased from Sigma-Aldrich (Sydney, NSW, Australia). Cisplatin-resistant EOC cell line IGROV1-cis was kindly provided by Prof. Jan H.M. Schellens (Netherlands Cancer Institute, AMS, Netherlands) (8). Immortalized primary ovarian epithelial cell line (HOSE) was obtained from Garvan Institute of Medical Research (Sydney, NSW, Australia). All cell culture reagents were supplied by Invitrogen (Melbourne, VIC, Australia). A2780, A2780-cis, IGROV1, IGROV1-cis, and SKOV-3 cell lines were cultured in RPMI-1640 medium supplemented with 10% fetal bovine serum (FBS), 50 U/mL penicillin, and 50 μg/mL streptomycin. Cisplatin was purchased from Sigma–Aldrich (Castle Hill, NSW, Australia). The stock solution of cisplatin was prepared in DMSO (Sigma–Aldrich, Castle Hill, NSW, Australia), while the working solution of cisplatin was prepared in cell culture medium and the final concentration of DMSO was 0.1%. 1 and 3.3 μM cisplatin were added to the medium every 2–3 passages for A2780-cis and IGROV1-cis cell lines, respectively. OV-90 and HOSE cell lines were maintained in a 1:1 mixture of MCDB 105 and 199 medium (Sigma-Aldrich) supplemented with 15% FBS, 50 U/mL penicillin, and 50 μg/mL streptomycin. All cell lines were maintained in a humidified incubator at 37°C and 5% CO_2_.

### Cell Viability Analysis

5 × 10^3^ cells were seeded in 96-well plates for 24 h and then treated with a gradient of concentrations of cisplatin. After 48 h incubation, cell viability was detected using 3-(4,5-dimethylthiazol-2-yl)-2,5-diphenyltetrazolium bromide (MTT, 0.5 mg/mL) and BIO-TEK microplate reader (Bio-Rad, Hercules, CA, USA) at 562 nm wavelength. IC_50_ values were calculated.

### Protein Samples for Proteomics

A2780, A2780-cis, IGROV-1, and IGROV-1-cis cells with 80% confluence were harvested into cold 1 × PBS by a scraper and washed three times by 1 × PBS. The protein samples were prepared according to our previous study (9). A total of 100 μg protein was precipitated in 100% ice-cold acetone at 1:4 (v/v), incubated with trypsin at an enzyme/substrate ratio of 1:50 (w/w) at 37°C overnight, and then eluted using Pierce™ C18 Spin Tips (ThermoFisher Scientific, VIC, Australia).

### Label-Free LC-MS/MS-Based Proteomics

Label-free LC-MS/MS-based proteomics was performed using an LTQ Orbitrap Velos ETD (Thermo Scientific, USA) at Bioanalytical Mass Spectrometry Facility, UNSW Sydney as described in our previous report (9). Briefly, 0.5 μL sample was loaded into the micro C18 pre-column (300 μm × 5 mm, Dionex, Scoresby, VIC, Australia) with Buffer A (98% H_2_O, 2% CH_3_CN, and 0.1% formic acid) at 10 μL/min, followed by a fritless nano-column (75 μm i.d × 15 cm) containing reverse phase C18 media (3 μm, 200 Å Magic, Michrom Bioresources). Samples were eluted by a linear gradient of Buffer A to Buffer B (98% CH_3_CN, 2% H_2_O, 0.1% formic acid) at 0.25 μL/min in 140 min. High voltage (2,000 V) was applied to low volume tee (Upchurch Scientific, Oak Harbor, WA, USA) and the column tip positioned 0.5 cm from the heated capillary (280°C) of an Orbitrap Velos mass spectrometer (Thermo Electron, Bremen, Germany). Positive ions were generated by electrospray and the Orbitrap was operated in a data-dependent acquisition mode. A survey scan 350–1750 m/z was acquired in the Orbitrap at a resolution of 30,000 with lock-mass enabled. Up to 10 most abundant ions (>5,000 counts) with charge states +2 to +4 were isolated and fragmented within the linear ion trap using collisionally induced dissociation with an activation q = 0.25 and activation time of 30 ms at a target value of 30,000 ions. The m/z ratios selected for MS/MS were dynamically excluded for 30 s.

### Protein Quantification Analysis

Data of MS peaks were analyzed using Progenesis QI software V4 (Waters, NC, USA). Ion intensity maps were aligned to a reference sample and ion matching was performed by aligning consistent ion m/z and retention times. Protein identification was achieved by Mascot Daemon (Matrix Science, London, UK). Type I errors were controlled using 1% False Discovery Rate. Peptide intensities were normalized against the total intensity (a sample-specific log-scale abundance ratio scaling factor) and compared by one-way ANOVA with *post-hoc* test. *P* < 0.05 was considered as statistically significant.

### Western Blot (WB)

Total and nuclear proteins were extracted, run on NuPAGE Novex 4–12% Bis-Tris gel (Life Technologies, VIC, Australia), and then transferred to polyvinylidene difluoride membrane. After blocking with 5% bovine serum albumin (BSA), the membrane was incubated with different primary antibodies (Table 1) at 4°C overnight and then incubated with HRP-conjugated secondary antibodies. Protein bands were detected using an Enhanced Chemiluminescence kit (Pierce Chemical Co, USA) and ImageQuant LAS4000 system (GE Healthcare, USA). β-tubulin and Lamin B were used as the loading control in this study.

**Table 1:** Antibodies for immunofluorescence (IF) staining and western blot (WB).

**Antibodies**	**Sources**	**Types**	**Dilution ratio**	**Incubation time (min) and temperature**
Rabbit anti-human C1ORFF77 (CHTOP)	Abcam	MAb	1:500 (WB) 1:100 (IF)	O/N, 4°C
Rabbit anti-human uncleaved PARP-1	Abcam	MAb	1:1,000 (WB)	O/N, 4°C
Rabbit anti-human cleaved PARP-1	Abcam	PAb	1:500 (WB)	O/N, 4°C
Rabbit anti-human active Caspase 3	Abcam	PAb	1:500 (WB)	O/N, 4°C
Rabbit anti-human active Caspase 7	Abcam	PAb	1:500 (WB)	O/N, 4°C
Rabbit anti-human Bax	Abcam	PAb	1:500 (WB)	O/N, 4°C
Rabbit anti-human Bcl-2	Abcam	MAb	1:1,000 (WB)	O/N, 4°C
Rabbit anti-human Bcl-xL	Abcam	PAb	1:500 (WB)	O/N, 4°C
Mouse anti-human CD44	Abcam	MAb	1:1,000 (WB) 1:100 (IF)	O/N, 4°C
Rabbit anti-human CD105	Abcam	MAb	1:1,000 (WB) 1:100 (IF)	O/N, 4°C
Rabbit anti-human c-Kit	Abcam	PAb	1:1,000 (WB) 1:100 (IF)	O/N, 4°C
Rabbit anti-human Snail	Abcam	PAb	1:1,000 (WB) 1:100 (IF)	O/N, 4°C
Mouse anti-human Oct-4	Abcam	MAb	1:1,000 (WB) 1:100 (IF)	O/N, 4°C
Rabbit anti-human Nanog	Abcam	PAb	1:1,000 (WB) 1:100 (IF)	O/N, 4°C
Rabbit anti-human Sox-2	Abcam	PAb	1:1,000 (WB) 1:100 (IF)	O/N, 4°C
Rabbit anti-human Lamin B	Abcam	MAb	1:1,000 (WB)	O/N, 4°C
Rabbit anti-human β-tubulin	Sigma-Aldrich	MAb	1:3,000 (WB)	O/N, 4°C
Goat anti-human ALDH1	Santa Cruz	PAb	1:200 (WB, IF)	O/N, 4°C
Goat anti-rabbit IgG-HRP	Santa Cruz	IgG	1:3,000 (WB)	60, RT
Goat anti-mouse IgG-HRP	Santa Cruz	IgG	1:3,000 (WB)	60, RT
Donkey anti-goat IgG-HRP	Santa Cruz	IgG	1:3,000 (WB)	60, RT
Goat anti-mouse Alexa Fluor® 488 Dye	Invitrogen	IgG	1:1,000 (IF)	45, RT
Goat anti-rabbit Alexa Fluor® 488 Dye	Invitrogen	IgG	1:1,000 (IF)	45, RT
Donkey anti-goat Alexa Fluor® 488 Dye	Invitrogen	IgG	1:1,000 (IF)	45, RT

*MAb, monoclonal antibody; PAb, polyclonal antibody; O/N, overnight; RT, room temperature*.

### Immunofluorescence (IF)

1 × 10^5^ cells were seeded on glass cover-slips for 24 h. After washing with Tris-buffered saline (TBS), cells were fixed using methanol and then incubated with 10% goat serum (donkey serum for ALDH1) for 30 min at room temperature (RT). These cells were further incubated with different primary antibodies (Table 1) at 4°C overnight and then incubated with Alexa Fluro-488 goat anti-rabbit IgG, Alexa Fluro-488 goat anti-mouse IgG, or Alexa Fluro-488 donkey anti-goat IgG (1:1,000 dilution) for 1 h at RT. Propidium iodide (PI, 0.2 mg/L) was used for nuclear staining. IF was photographed using an inverse fluorescence microscope (Zeiss, Australia).

### Immunohistochemistry (IHC)

Paraffin-embedded human tissue microarray (TMA) slide (Cat#: ov1005b, USA Biomax, Derwood, MD, USA) was deparaffinized in xylene, rehydrated in ethanol, immersed in Target Retrieval Solution (Dako, CA, USA), and heated in boiling water for 15 min. Then slides were incubated with a rabbit anti-human CHTOP monoclonal antibody at 1:100 dilution for 1 h at RT. After incubation with horseradish peroxide-conjugated secondary antibody (1:300 dilution) for 30 min at RT, the immunoreactivity was initiated using Liquid DAB+ Substrate Chromogen System (Dako). Finally, the slides were counterstained with Harris Hematoxylin (ThermoFisher Scientific) for 1 min and photographed immediately. Control slides were treated identically and stained with an isotype-matched non-specific immunoglobulin. The detailed information of TMA cases was shown in Supplementary Table 1.

### Immunostaining Assessment

Staining intensity score (0–3) in EOC cell lines and TMA tissue were assessed using an inverse fluorescence microscope (Zeiss, Australia) or a light microscope (Leica, Germany), respectively. The criteria used for assessment were 0 (negative, < 25%), 1 (weak, 25–50%), 2 (moderate, 50–70%), 3 (strong, > 75%). Evaluation of tissue staining was performed independently by four observers (XF, XB, JN, and YL). All specimens were scored blind and an average of scores was taken finally.

### Bioinformatic Analysis

Kaplan–Meier analysis of PFS with log-rank tests for CHTOP expression was performed using Kaplan-Meier plotter (www.kmplot.com/analysis) with 2017 version database [GSE65986 (*n* = 55), GSE14764 (*n* = 80), and all ovarian cancer patients (*n* = 1,435)]. The median CHTOP expression value was set at 2,652 (expression range 1,445–6,073). Gene expression and PFS data were obtained from Gene Expression Omnibus and The Cancer Genome Atlas (10).

### Small Interfering RNA (siRNA) Transfection

CHTOP siRNA or control siRNA (scr) were obtained from Life Technologies. CHTOP knockdown (KD) was performed using Lipofectamine RNAiMAX transfection reagent (Life technologies) and siRNAs according to manufacturer's protocol. After incubation with siRNAs for 72 h, cells were ready for following experiments.

### Quantitative Real-Time PCR (qRT-PCR)

Total RNA was extracted using the RNAeasy kit (Qiagen, VIC, Australia) according to manufacturer's instructions. cDNA was synthesized using the iScript™ Reverse Transcription Supermix (Bio-rad, CA, USA). CHTOP primers were purchased from Bio-rad (Assay ID: qHsaCED0057444). QRT-PCR mixture containing 25 ng cDNA, 1 μL CHTOP primers and 10 μL SsoAdvanced universal SYBR® Green Supermix was run in CFX96™ Real-Time PCR Detection Systems (Bio-rad) according to the manufacturer's protocol. Positive PCR control, reverse transcription control, and DNA contamination control were run simultaneously as quality controls for the study. The threshold cycle was obtained in CFX Manager™ Software V3.1 (Bio-rad) and normalized against the reference gene GAPDH. The relative fold changes of CHTOP were calculated using the 2^−ΔΔ^CT method.

### Apoptosis Analysis

Cell apoptosis was analyzed using flow cytometry. Briefly, cells were seeded in a 75 cm^2^ flask for 48 h and treated with vehicle control or cisplatin for 48 h. Then, cells were collected and incubated with Annexin V-FITC for 15 min at room temperature in the dark. Analysis was performed at FACSCanto II Flow Cytometer (BD Biosciences).

### TUNEL Assay

2 × 10^5^ cells were cultured in 25 cm^2^ flasks for 24 h and then treated with vehicle control or cisplatin for 48 h. Apoptosis from different groups of cells was detected using TUNEL assay kit (R&D systems, MN, USA) according to manufacturer's protocol and examined using a light microscope (Leica, Germany) at 400 × magnification.

### Sphere Formation Assay

Cells were seeded in an ultra-low attachment round bottom 6-well plate (Sigma–Aldrich) at a density of 2,000 per well using serum-free DMEM/F12K media supplemented with 4 μg/mL insulin (Sigma-Aldrich), 1 × B27 (Gibco, VA, USA), 20 ng/mL EGF (Sigma-Aldrich), and 20 ng/mL bFGF (Sigma-Aldrich). Spheres were counted after 5 days and the diameter of each sphere was measured by an inverted phase microscope (CK-2, Olympus, Tokyo, Japan) fitted with an ocular eyepiece.

### Matrigel Invasion Assay

Cell invasion ability was evaluated using Matrigel invasion plates (BD Bioscience, Australia). Briefly, 2 × 10^4^ cells in 500 μL serum-free RPMI-1640 medium were added to each chamber, while 750 μL complete medium was added to each well. Cells were incubated for 48 h and then stained with Diff-Quick staining kit (Allegiance Healthcare Corp, USA). The number of cells that invade through Matrigel or control chamber in five high power fields was counted using a light microscope (Leica, Germany). The invasion ability was calculated as [(mean number of cells invading through Matrigel chamber) / (mean number of cells migrating through control chamber)] × 100%.

### Wound-Healing Assay

Cells were seeded in a 6-well plate at the density of 3 × 10^5^ per well for 24 h. Then, a sterile 200 μL yellow pipette tip was used to scrape the cell monolayers, causing a straight wound. Cell debris was removed through washing with 1 × PBS and then the culture medium was added. Representative images were obtained at 0, 48, and 96 h after the scratch, respectively, using a light microscope (Leica, Germany) at 40 × magnification.

### Statistical Analysis

All numerical data were expressed as mean ± standard deviation (SD) from at least three independent experiments. Data were compared using one-way ANOVA with *post-hoc* Dunnett's test. *P* < 0.05 was considered statistically significant. All graphs were generated using GraphPad® Prism 7 (GraphPad, CA, USA).

## Results

### CHTOP Was Highly Expressed in Cisplatin-Resistant and Metastatic EOC Cells and Associated With Poor Progression-Free Survival in Patients

In this study, the cell viability under cisplatin treatment was first detected using MTT assay. As shown in Figure 1A, A2780-cis and IGROV1-cis cells showed enhanced resistance to cisplatin as compared to their parental cells (A2780 and IGROV1). The IC_50_ for A2780-cis and IGROV1-cis cells were 32.6 μM and 51.7 μM, respectively. Thus, the concentration of cisplatin used for the following studies is half of the IC_50_ of the cell line (16 μM for A2780-cis and 26 μM for IGROV1-cis). Using LC-MS/MS-based label-free proteomics, 56 proteins that were differentially expressed in EOC-cis cell lines (A2780-cis and IGROV1-cis) compared with their parental cell lines were identified (Figure 1B). Thereinto, CHTOP was selected for further investigation. Results from WB and IF showed that CHTOP was highly expressed in EOC-cis and metastatic cells (SKOV3 and OV90) as compared to sensitive cells and normal epithelial ovarian cells (HOSE) (Figure 1C). IF staining scores were summarized in Supplementary Table 2.

**Figure 2:**
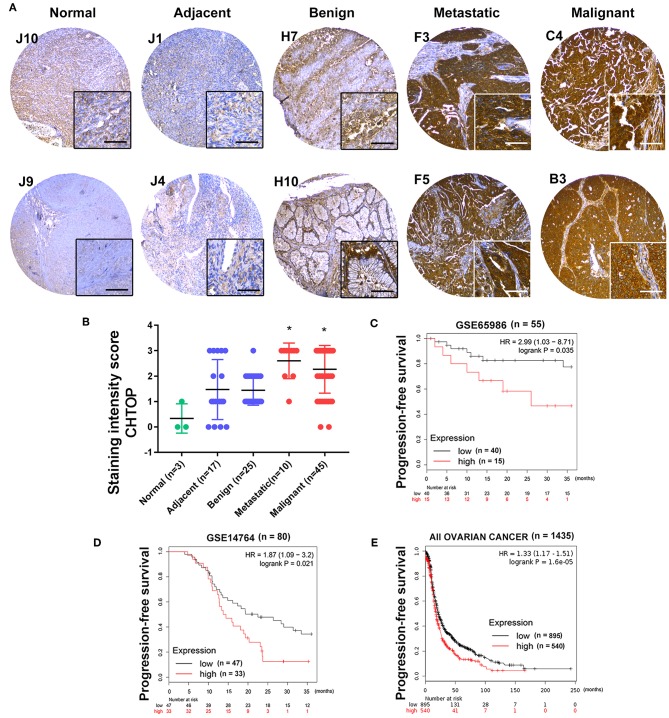
CHTOP was highly expressed in human metastatic and malignant ovarian cancer tissues and associated with a poor PFS in patients. **(A)** Representative IHC images from human ovarian cancer TMA showing a significantly higher expression of CHTOP in metastatic and malignant ovarian cancer tissues as compared to normal, adjacent, or benign tissues. IHC images were photographed at 100 × and 400 × magnification. Brown represents positive CHTOP staining, while blue represents nucleus. **(B)** The average staining intensity scores of CHTOP among different tissues from human ovarian cancer TMA were compared. *P < 0.05 vs. normal ovary tissues. **(C–E)** Higher expression of CHTOP was associated with a lower PFS rate in ovarian cancer patients.

Furthermore, a higher expression of CHTOP was observed in metastatic and malignant ovarian cancer tissues as compared to the normal ovary and adjacent tissues which displayed weak staining of CHTOP (Figure 2A). The staining intensity score of CHTOP in malignant or metastatic ovarian cancer tissues was significantly higher than that in normal, adjacent, or benign tissues (Figure 2B). Moreover, Kaplan–Meier analysis showed that the high expression of CHTOP in ovarian cancer tissues was associated with a lower PFS rate in patients (Figures 2C–E). These results suggest that the overexpression of CHTOP is associated with EOC cisplatin resistance and metastasis.

### Knockdown of CHTOP Enhanced Cisplatin-Induced Apoptosis in Cisplatin-Resistant EOC Cells

**Figure 3:**
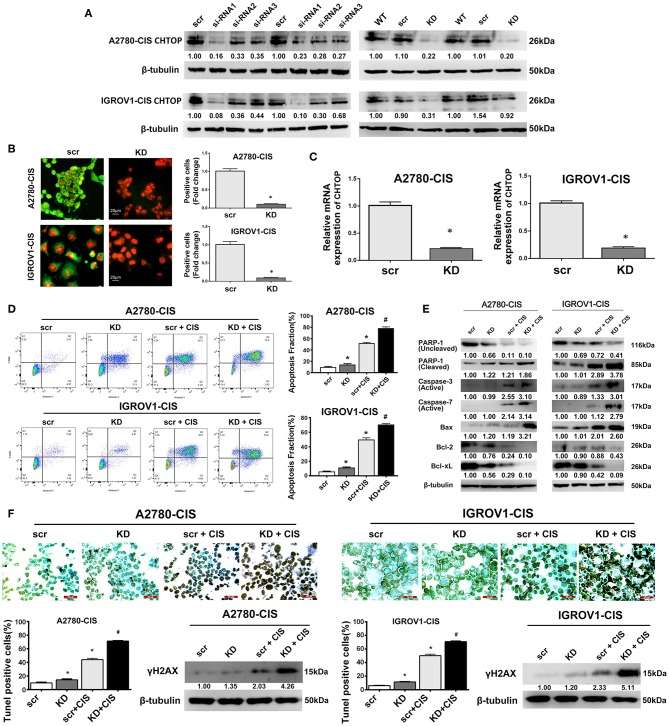
CHTOP KD enhanced cisplatin-induced apoptosis in cisplatin-resistant EOC cells. **(A)** The expression of CHTOP in two cisplatin-resistant cell lines was significantly decreased by CHTOP siRNA1. **(B)** The number of CHTOP-positive cells in two cisplatin-resistant cell lines was significantly decreased by CHTOP KD. IF images were photographed at 400 × magnification. Green fluorescence represents CHTOP, while red fluorescence represents nucleus. **(C)** The mRNA expression of CHTOP in two cisplatin-resistant cell lines was significantly decreased by CHTOP KD. **(D)** Cell apoptosis was analyzed by flow cytometry. Either CHTOP KD or cisplatin treatment can induce apoptosis in cisplatin-resistant EOC cells, while the combination treatment with CHTOP KD and cisplatin can further enhance this effect. **(E)** Results from WB analysis showed that CHTOP KD can further enhance cisplatin-induced activation of apoptosis signaling. **(F)** TUNEL assay was used to detect DNA double strand breaks induced by CHTOP KD at the presence or absence of cisplatin. Compared with single cisplatin treatment, TUNEL-positive cells were significantly increased by combination treatment. This result is in line with the markedly increased expression of γH2AX. Images for TUNEL assay were photographed at 400 × magnification. Data were expressed as mean ± SD (*n* = 3). *P < 0.05 vs. control group, ^#^*P* < 0.05 vs. cisplatin treatment group.

In this study, CHTOP-specific siRNA that can effectively knock down the protein expression of CHTOP was screened. As shown in Figure 3A, siRNA1 [sequence (5′-3′): sense, CCUUGAGAUAACAGAUGAGtt, antisense, CUCAUCUGUUAUCUCAAGGtt] showed significantly effective KD effect in EOC-cis cell lines as compared to siRNA2 and siRNA3. Results from IF showed that CHTOP KD by siRNA1 could significantly reduce CHTOP protein expression in EOC-cis cell lines (Figure 3B). IF staining scores were summarized in Supplementary Table 3. In addition, qRT-PCR analysis further confirmed that siRNA1 could effectively reduce the mRNA expression of CHTOP (Figure 3C). These results suggest that CHTOP expression can be effectively knocked down by CHTOP-specific siRNA1. Thus, siRNA1 was used for following experiments.

To investigate the role of CHTOP in EOC chemoresistance, cell apoptosis after CHTOP KD in the presence or absence of cisplatin was analyzed. As shown in Figure 3D, CHTOP KD significantly increased the apoptotic fraction of cisplatin-resistant EOC cells. More importantly, compared with treatment with cisplatin and scr, combination treatment with cisplatin and si-CHTOP further increased the apoptotic fraction of cisplatin-resistant EOC cells (Figure 3D). Additionally, results from WB showed that CHTOP KD slightly increased the protein expressions of cleaved PARP-1 and Bax and decreased the protein expressions of Bcl-2 and Bcl-xL as compared to scr group, while treatment with cisplatin and si-CHTOP significantly increased the protein expressions of cleaved PARP-1, active Caspase-3, active Caspase-7, and Bax and reduced the expressions of Bcl-2 and Bcl-xL compared with the treatment with scr and cisplatin (Figure 3E). The similar results were also obtained from TUNEL assay. As shown in Figure 3F, compared with scr group, CHTOP KD markedly increased TUNEL-positive cells in both cisplatin-resistant EOC cell lines. This difference was more significant in the presence of cisplatin. It was shown that CHTOP KD and cisplatin significantly increased TUNEL-positive cells as compared to the treatment with scr and cisplatin (Figure 3F). Consistently, the protein expression of γH2AX, a marker for DNA double-strand breaks, was significantly induced by the treatment with si-CHTOP and cisplatin as compared to the treatment with scr and cisplatin (Figure 3F). These results suggest that CHTOP KD sensitized cisplatin-resistant EOC cells to cisplatin through enhancing cisplatin-induced apoptosis.

### Knockdown of CHTOP Reduced the Stemness of Cisplatin-Resistant EOC Cells

**Figure 4:**
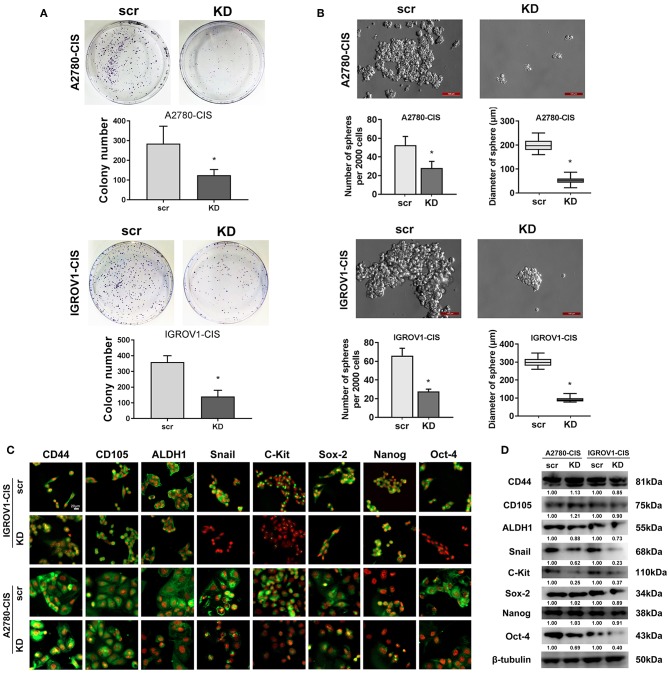
CHTOP KD reduced the stemness of cisplatin-resistant EOC cells. **(A)** colony formation assay was used to evaluate the effect of CHTOP KD on the colony formation ability of cisplatin-resistant EOC cells. As shown, CHTOP KD significantly decreased the number of colonies in both cisplatin-resistant EOC cell lines. **(B)** Sphere formation assay was employed to evaluate the effect of CHTOP KD on the tumorigenic ability of two cisplatin-resistant EOC cell lines. As shown, CHTOP KD significantly reduced the number of spheres as well as the diameter of developed spheres in both cisplatin-resistant EOC cell lines. Images were obtained at 200 × magnification. **(C)** The expressions of ALDH1, Snail, c-Kit, and Oct-4 were significantly decreased by CHTOP silence in two cisplatin-resistant EOC cell lines. IF images were photographed at 400 × magnification. Green fluorescence represents positive marker staining, while red fluorescence represents nucleus. **(D)** The protein expressions of ALDH1, Snail, c-Kit, and Oct-4 in two cisplatin-resistant EOC cell lines were significantly decreased by CHTOP silence. Data were expressed as mean ± SD (*n* = 3). *P < 0.05 vs. control group.

As stemness is closely associated with the chemoresistance, we next investigated the effect of CHTOP KD on the stemness of cisplatin-resistant EOC cell lines. Results from colony formation assay showed that CHTOP KD significantly decreased the colony number of cisplatin-resistant EOC cells (Figure 4A). Further results from sphere formation assay showed that the sphere number and diameter in both cisplatin-resistant EOC cell lines were much higher than that in scr group (Figure 4B). The average sphere diameters for A2780-cis and IGROV1-cis cells treated with were around 200 and 300 μm, respectively, while the average sphere diameters for A2780-cis and IGROV1-cis in CHTOP KD groups were ~ 50 and 100 μm, respectively (Figure 4B).

To further investigate the association of CHTOP with cisplatin-resistant EOC cell stemness, the protein expressions of several representative ovarian cancer stem cell (CSC) markers after CHTOP KD were detected in this study. As shown in Figure 4C, results from IF showed that the protein expressions of ALDH1, Snail, c-Kit, and Oct-4 were decreased by CHTOP KD as compared to the scr group. The IF staining scores were summarized in Supplementary Table 4. Similar results were also obtained from WB analysis in which CHTOP KD was found to significantly reduce the protein expressions of ALDH1, Snail, c-Kit, and Oct-4 in cisplatin-resistant EOC cells (Figure 4D). However, there is no significant difference in CD44, CD105, Sox-2, and Nanog between scr group and CHTOP KD group (Figure 4D). In addition, the nuclear protein expressions of Sox-2, Nanog, and Oct-4 were also detected in this study. As shown in Supplementary Figure 1, the nuclear translocation of Oct-4 was markedly decreased by CHTOP KD in both cisplatin-resistant EOC cell lines, whereas no significant difference was found in Sox-2 and Nanog. These results suggest that CHTOP KD can affect the translocation of some CSC markers such as Oct-4.

### Knockdown of CHTOP Disrupted the Invasion and Migration Ability of Cisplatin-Resistant EOC Cells

**Figure 5:**
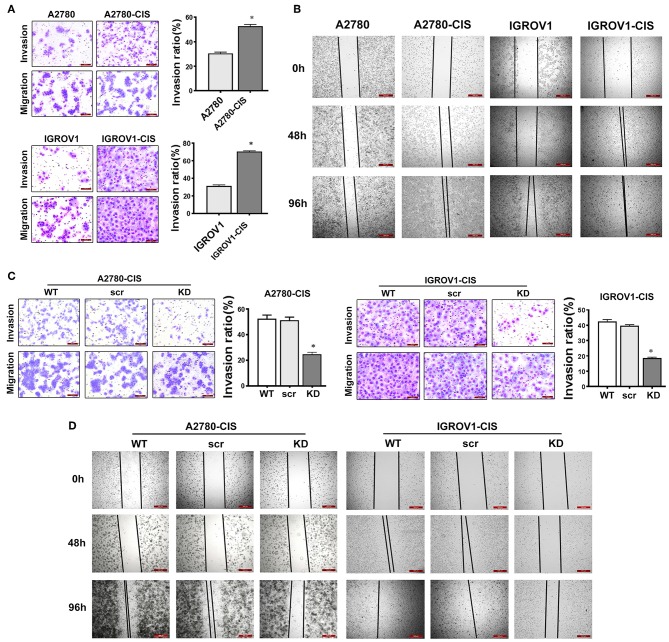
CHTOP KD disrupted the invasion and migration ability of cisplatin-resistant EOC cells. **(A)** The invasion ability of cells was examined using Matrigel invasion assay. Cisplatin-resistant EOC cell lines had higher invasive potential compared with their parental cell lines. **(B)** The migration potential of cells was tested using wound-healing assay. Cisplatin-resistant EOC cell lines had higher migration potential as compared to their parental cell lines. **(C)** CHTOP KD significantly disrupted the invasion ability of cisplatin-resistant EOC cell lines. **(D)** CHTOP KD significantly disrupted the migration ability of cisplatin-resistant EOC cell lines. Images for invasion assay were photographed at 200 × magnification, while wound-healing images were photographed at 40 × magnification. Data were expressed as mean ± SD (*n* = 3). *P < 0.05 vs. control group.

Since high expression of CHTOP was found in metastatic human ovarian cancer cell lines and tissues, we then also detected the invasion and migration ability of cisplatin-resistant EOC cells. As shown in Figure 5A, cisplatin-resistant cell lines were characterized by an increased invasive potential as compared to their parental cell lines. The average invasion rate for A2780-cis and IGROV1-cis were ~ 52 and 71%, respectively, whereas the average invasion rate for A2780 and IGROV1 were only around 30% (Figure 5A). Besides, results from wound-healing assay showed a much higher wound closure rate in cisplatin-resistant EOC cell lines (Figure 5B). These results suggest that cisplatin-resistant cells had higher metastatic potential compared with their parental cells.

To investigate the role of CHTOP in the metastasis of cisplatin-resistant EOC cells, Matrigel invasion and wound-healing assays were employed to evaluate the invasion and migration ability of these cells after CHTOP KD. Results from Figure 5C showed that CHTOP KD significantly decreased the invasion ability of cisplatin-resistant EOC cells compared with wild-type (WT) and scr group. Also, CHTOP KD significantly reduced the migration rate of both A2780-cis and IGROV1-cis cells as compared to WT and scr group (Figure 5D). These results suggest that CHTOP KD can disrupt the metastatic ability of cisplatin-resistant EOC cells.

## Discussion

**Figure 6:**
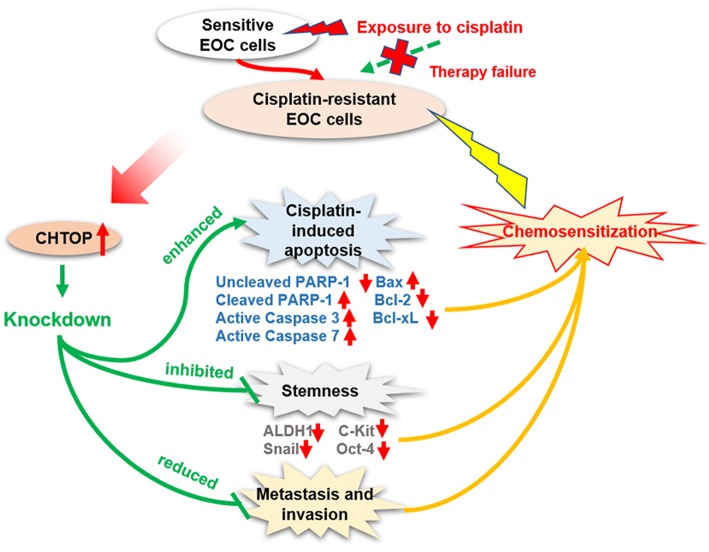
Diagram showing the proposed role of CHTOP in EOC chemoresistance. CHTOP inhibition can restore the sensitivity of cisplatin-resistant EOC cells toward cisplatin by enhancing the apoptosis signaling, reducing the stemness, and disrupting the invasion/migration ability.

Chemoresistance is a major clinical obstacle in EOC treatment. The molecular mechanisms of resistance to platinum compounds are still not fully elucidated. Hence, there is an urgent need to understand the mechanism underlying cisplatin-resistance and to identify novel biomarkers to predict the response to cisplatin-based therapy or to develop novel strategies aimed at improving the clinical outcome of EOC patients. In the current study, CHTOP, as a novel biomarker, was identified from our proteomics study, and its role in EOC chemoresistance was uncovered. The summary of our findings is shown in Figure 6.

Firstly, our results showed that CHTOP was highly expressed in cisplatin-resistant and metastatic EOC cell lines, and no positive expression was found in normal epithelial ovarian cells, indicating that CHTOP may play an essential role in EOC chemoresistance and metastasis. More importantly, we also found a much higher expression of CHTOP in human metastatic and malignant EOC tissues as compared to normal ovary tissues, adjacent tissues, or benign tumor tissues, suggesting that CHTOP might be a potential specific target for advanced ovarian cancer. Furthermore, we demonstrated that the high expression of CHTOP was associated with a lower PFS rate in ovarian cancer patients using online survival dataset. These findings indicate that CHTOP is an important molecular target associated with EOC chemoresistance, metastasis, and prognosis, which deserves to be further investigated for its therapeutic value.

Cisplatin-resistant cancer cells usually show a multidrug-resistant phenotype, which might be developed by several mechanisms, including decreased drug uptake, increased drug efflux, activation of detoxifying systems, activation of DNA repair, and evasion of drug-induced apoptosis (11, 12). Although the exact mechanisms underlying the cytotoxic effect of platinum agents were not fully elucidated, the irreversible DNA damage induced by platinum agents often leads to an activation of the apoptotic pathway. Thus, the cisplatin resistance is thought to be a consequence of its inability to induce apoptosis. Results from flow cytometry analysis demonstrated that combination treatment with si-CHTOP and cisplatin could induce more apoptosis in cisplatin-resistant EOC cells than mono-treatment with cisplatin, suggesting that CHTOP may play an important role in protecting cisplatin-resistant EOC cells against apoptosis. Moreover, the enhanced activation of apoptosis pathway (increased protein expressions of cleaved PARP-1, active Caspase-3, active Caspase-7, and Bax and decreased protein expressions of Bcl-2 and Bcl-xL) in the combination treatment group further supported the findings from flow cytometry analysis.

It is reported that intracellular DNA double-strand break resulted from the crosslink is the principal cytotoxic mechanism of cisplatin (11). Thus, in the current study, DNA double-strand breaks were measured using TUNEL assay as well as γH2AX. TUNEL assay is a highly specific and sensitive method for monitoring both DNA double-strand break initiation and resolution (13), while γH2AX acts as a highly sensitive marker of DNA damage for predicting potential cell chemosensitivity to interstrand crosslinking agents (14). It has been used to assess DNA damage in different cancers and to evaluate the treatment response to cisplatin-based therapies in EOC (15–17). In our results, we observed that TUNEL-positive cells were significantly increased by combination treatment with si-CHTOP and cisplatin compared with the mono-treatment of cisplatin. Consistently, γH2AX protein expression was also markedly increased in si-CHTOP and cisplatin treatment group, suggesting that the combination treatment induced more DNA double-strand breaks, which is in line with the higher percentage of apoptotic cells caused by the combination therapy.

CSCs are becoming recognized to take more responsibility for the malignant behaviors of the tumor (18), such as evading chemotherapeutic challenges and maintaining the continuity of a tumor. The contribution of CSCs to cisplatin resistance has been uncovered in many cancer types (19, 20). It was demonstrated that cisplatin treatment could enrich CSCs in ovarian cancer (21, 22), and these ovarian CSCs could survive cisplatin cytotoxicity through the enhancement of miR-93 expression (23). Due to the critical role of CSCs in the regulation of EOC chemoresistance, we hypothesized that CHTOP might be associated with CSCs in cisplatin-resistant EOC cells. To validate our hypothesis, we examined the sphere formation ability of cisplatin-resistant EOC cells after CHTOP KD using sphere formation assay. It was reported that this assay is a golden *in vitro* method for isolating cancer cells with conserved stemness determinants (24), which best mimics the process of enriching and proliferating of CSCs. Collura et al. (25) indicated that the sphere model derived from cancer cell lines possesses CSC features that may have clinical relevance for drug resistance and disease relapse. In the current study, we found that CHTOP KD can significantly reduce the number of spheres in two cisplatin-resistant EOC cell lines as compared to the scr groups, indicating that the stemness of cisplatin-resistant EOC cells is closely associated with CHTOP expression. Furthermore, as CSC marker expression is crucial evidence for evaluating cancer stemness, we also investigated which CSC markers were affected by CHTOP KD. Our results showed that CHTOP KD obviously reduced the expressions of ALDH1, Snail, c-Kit, and Oct-4, further confirming that CHTOP may be a potential biomarker for EOC chemoresistance-associated stemness.

Another important finding from this study is the enhanced invasion and migration ability in cisplatin-resistant EOC cells compared with their parental cells, suggesting that chemoresistant EOC cells are characterized by a higher metastatic potential. This is consistent with our observations in human TMA that showed a higher staining score of CHTOP in metastatic lesions compared with normal ovary tissues. In contrast, the invasion and migration potential of cisplatin-resistant EOC cells was greatly weakened by CHTOP KD, revealing an association of CHTOP with metastasis in chemoresistant EOC cells.

To the best of our knowledge, the clinical relevance of CHTOP has never been reported before. Based on our current results, it is worthwhile deeply investigating the regulative role of CHTOP in cancer therapeutic resistance and metastasis. Animal study is warranted to test whether CHTOP KD can inhibit tumor growth and promote chemosensitization. In summary, we demonstrate for the first time that CHTOP is a novel biomarker associated with chemoresistance, stemness, and metastasis in chemoresistant EOC which is promising in overcoming EOC chemoresistance as a molecular target and predicting ovarian cancer prognosis as a clinical indicator.

## Data Availability

The datasets used and/or analyzed in this study are available from the corresponding author on reasonable request.

## Author Contributions

YL: conceived and designed the experiments. XF, VW, JN, and JB: performed the experiments. XB and XF: analyzed the data. XB and YL: wrote the paper. XB, JN, YL, YZ, and PG: revised the paper.

### Conflict of Interest Statement

The authors declare that the research was conducted in the absence of any commercial or financial relationships that could be construed as a potential conflict of interest.
