# Supplementary material for: CHTOP in Chemoresistant Epithelial Ovarian Cancer: A Novel and Potential Therapeutic Target
Source: Front Oncol. 2019 Jun 27;9:557. doi: 10.3389/fonc.2019.00557 (PMC6660285; doi:10.3389/fonc.2019.00557)
Supplement: Supplementary file 1 [file Data_Sheet_1.pdf]

Supplementary Figure 1. CHTOP knockdown reduced the stemness of cisplatin-resistant EOC cells. The nuclear protein expressions of Sox-2, Nanog, and Oct-4 were detected by WB. The nuclear protein expression of Oct-4 was significantly decreased by CHTOP KD in two cisplatin-resistant EOC cell lines, while no significant difference was found in Sox-2 and Nanog. Lamin B was used as the loading control.

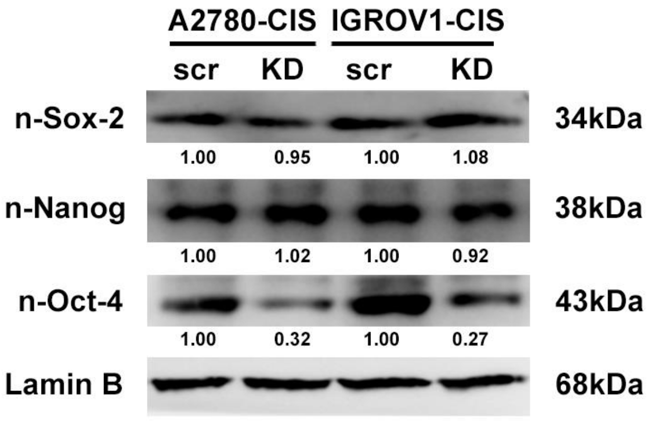

Supplementary Table 1. The information of tissue microarray cases used in this study.

Position	Age	Site	Pathology diagnosis	Grade	Stage	Type	Tissue ID.	Score
A1	65	Ovary	Serous papillary cystadenocarcinoma	1	I	Malignant	Fov041172	1
A2	38	Ovary	Serous papillary cystadenocarcinoma	1	IV	Malignant	Fov010473	2
A3	51	Ovary	Serous papillary cystadenocarcinoma	1	IIIc	Malignant	Fov010563	3
A4	22	Ovary	Serous papillary cystadenocarcinoma	1	IIb	Malignant	Fov010682	2
A5	48	Ovary	Serous papillary cystadenocarcinoma	1	I	Malignant	Fov010719	1
A6	26	Ovary	Serous papillary cystadenocarcinoma	1	IIIc	Malignant	Fov010750	3
A7	25	Ovary	Serous papillary cystadenocarcinoma	1	I	Malignant	Fov010772	1
A8	50	Ovary	Serous papillary cystadenocarcinoma	1	II	Malignant	Fov010720	3
A9	26	Ovary	Serous papillary cystadenocarcinoma	1	Ic	Malignant	Fov010378	2
A10	47	Ovary	Serous papillary cystadenocarcinoma	1	I	Malignant	Fov010394	3
B1	58	Ovary	Serous papillary adenocarcinoma with necrosis	2	I	Malignant	Fov020002	0
B2	57	Ovary	Serous papillary cystadenocarcinoma	2	Ic	Malignant	Fov010012	3
**B3**	**51**	**Ovary**	**Serous papillary adenocarcinoma**	**2**	**Ia**	**Malignant**	**Fov031536**	**3**
B4	52	Ovary	Serous papillary	2	II	Malignant	Fov010892	3

			cystadenocarcinoma					
B5	54	Ovary	Serous papillary adenocarcinoma	2	IIIc	Malignant	Fov010137	3
B6	33	Ovary	Serous papillary adenocarcinoma	2	I	Malignant	Fov010873	3
B7	56	Ovary	Serous papillary adenocarcinoma	3	II	Malignant	Fov020045	3
B8	41	Ovary	Serous papillary adenocarcinoma	2	I	Malignant	Fov020059	3
B9	46	Ovary	Serous papillary adenocarcinoma	2	IIIc	Malignant	Fov010784	3
B10	46	Ovary	Serous papillary adenocarcinoma	2	IIIc	Malignant	Fov010659	2
C1	57	Ovary	Serous adenocarcinoma	2	IIIc	Malignant	Fov010660	1
C2	75	Ovary	Serous adenocarcinoma	2--3	II	Malignant	Fov010663	2
C3	54	Ovary	Serous adenocarcinoma	3	IIIc	Malignant	Fov010165	3
**C4**	**49**	**Ovary**	**Serous adenocarcinoma**	**3**	**II**	**Malignant**	**Fov010181**	**3**
C5	50	Ovary	Serous papillary adenocarcinoma	2	I	Malignant	Fov010255	3
C6	52	Ovary	Serous adenocarcinoma	3	II	Malignant	Fov020061	3
C7	47	Ovary	Serous adenocarcinoma	3	IIIc	Malignant	Fov010120	3
C8	34	Ovary	Mucinous adenocarcinoma	2	Ib	Malignant	Fov032024	1
C9	63	Ovary	Mucinous adenocarcinoma	1	Ia	Malignant	Fov032225	1
C10	69	Ovary	Mucinous adenocarcinoma	1	Ib	Malignant	Fov032427	1
D1	46	Ovary	Endometrioid adenocarcinoma	1--2	II	Malignant	Fov021172	2
D2	47	Ovary	Endometrioid adenocarcinoma	1--2	IIa	Malignant	Fov020498	1
D3	54	Ovary	Endometrioid adenocarcinoma	1--2	Ib	Malignant	Fov050375	2
D4	65	Ovary	Adenocarcinoma (sparse)	-	Ic	Malignant	Fov030008	3
D5	55	Ovary	Endometrioid adenocarcinoma	2	I	Malignant	Fov010903	3
D6	54	Ovary	Endometrioid adenocarcinoma	1	Ib	Malignant	Fov020834	3
D7	43	Ovary	Endometrioid adenocarcinoma	1	Ic	Malignant	Fov020079	2
D8	55	Ovary	Endometrioid adenocarcinoma with necrosis	1	I	Malignant	Fov020275	2
D9	53	Ovary	Endometrioid adenocarcinoma	3	IIa	Malignant	Fov020722	3
D10	50	Ovary	Endometrioid adenocarcinoma	2	IIIc	Malignant	Fov020728	3
E1	51	Ovary	Transitional cell carcinoma with necrosis	2	Ib	Malignant	Fov030556	0

E2	39	Ovary	Transitional cell carcinoma with necrosis	2	Ia	Malignant	Fov030140	3
E3	38	Ovary	Transitional cell carcinoma	2--3	I	Malignant	Fov050117	3
E4	66	Ovary	Transitional cell carcinoma with squamous metaplasia	3	Ia	Malignant	Fov021500	3
E5	53	Ovary	Transitional cell carcinoma	2--3	I	Malignant	Fov041293	3
E6	47	Mesentery	Metastatic serous papillary cystadenocarcinoma from ovary	1	-	Metastatic	Amt050102	3
E7	57	Epiploon	Metastatic serous papillary cystadenocarcinoma from ovary	1	-	Metastatic	Aom020070	3
E8	65	Epiploon	Metastatic serous papillary cystadenocarcinoma with calcification from ovary	1	-	Metastatic	Aom030020	3
E9	59	Mesentery	Metastatic serous papillary cystadenocarcinoma from ovary	2	-	Metastatic	Amt030119	2
E10	28	Epiploon	Metastatic serous adenocarcinoma with calcification from ovary	2	-	Metastatic	Aom020015	2
F1	64	Epiploon	Metastatic serous papillary cystadenocarcinoma from ovary	1	-	Metastatic	Aom020055	1
F2	50	Epiploon	Metastatic serous papillary cystadenocarcinoma from ovary	1	-	Metastatic	Aom030147	3
**F3**	**58**	**Epiploon**	**Metastatic adenocarcinoma from ovary**	**2**	**-**	**Metastatic**	**Aom020056**	**3**
F4	47	Abdominal cavity	Metastatic adenocarcinoma from ovary	2	-	Metastatic	Aac060296	3
**F5**	**49**	**Abdominal cavity**	**Metastatic adenocarcinoma from ovary**	**3**	**-**	**Metastatic**	**Aac060313**	**3**
F6	34	Ovary	Borderline serous papillary cystadenoma	-	-	Borderline	Fov032374	2
F7	34	Ovary	Borderline serous papillary cystadenoma	-	-	Borderline	Fov020057	2
F8	28	Ovary	Borderline serous papillary cystadenoma	-	-	Borderline	Fov010816	2

F9	22	Ovary	Borderline serous papillary cystadenoma	-	-	Borderline	Fov020491	1
F10	60	Ovary	Borderline serous papillary cystadenoma	-	-	Borderline	Fov040393	1
G1	50	Ovary	Borderline serous papillary cystadenoma	-	-	Borderline	Fov061125	1
G2	37	Ovary	Borderline mucinous papillary cystadenoma	-	-	Borderline	Fov021955	1
G3	62	Ovary	Serous cystadenoma	-	-	Benign	Fov010590	2
G4	70	Ovary	Serous cystadenoma	-	-	Benign	Fov031113	1
G5	49	Ovary	Serous cystadenoma	-	-	Benign	Fov030069	1
G6	16	Ovary	Serous cystadenoma	-	-	Benign	Fov010664	1
G7	34	Ovary	Serous cystadenoma	-	-	Benign	Fov031105	1
G8	22	Ovary	Serous cystadenoma	-	-	Benign	Fov030190	1
G9	19	Ovary	Mucinous cystadenoma	-	-	Benign	Fov010302	2
G10	17	Ovary	Mucinous cystadenoma	-	-	Benign	Fov021510	1
H1	41	Uterus	Mucinous cystadenoma (ovary tissue)	-	-	Benign	Fur010103	1
H2	26	Ovary	Mucinous cystadenoma	-	-	Benign	Fov010654	1
H3	22	Ovary	Mucinous cystadenoma	-	-	Benign	Fov010743	2
H4	38	Ovary	Mucinous cystadenoma	-	-	Benign	Fov030257	3
H5	47	Ovary	Mucinous cystadenoma	-	-	Benign	Fov030281	2
H6	70	Ovary	Mucinous cystadenoma	-	-	Benign	Fov020840	1
**H7**	**51**	**Ovary**	**Mucinous cystadenoma**	**-**	**-**	**Benign**	**Fov030041**	**1**
H8	29	Ovary	Mucinous cystadenoma (fibrous tissue and blood vessel)	-	-	Benign	Fov020504	2
H9	35	Ovary	Mucinous cystadenoma (ovary tissue)	-	-	Benign	Fov021918	2
**H10**	**18**	**Ovary**	**Mucinous cystadenoma (fibrous tissue and blood vessel)**	**-**	**-**	**Benign**	**Fov021988**	**1**
I1	30	Ovary	Cancer adjacent normal ovary tissue	-	-	Adjacent	Fov010414	3
I2	39	Ovary	Cancer adjacent normal ovary tissue	-	-	Adjacent	Fov021899	3
I3	29	Ovary	Cancer adjacent normal ovary tissue	-	-	Adjacent	Fov021900	1
I4	41	Ovary	Cancer adjacent normal ovary tissue	-	-	Adjacent	Fov031969	2
I5	62	Ovary	Cancer adjacent	-	-	Adjacent	Fov032421	3

			normal ovary tissue					
I6	63	Ovary	Cancer adjacent normal ovary tissue	-	-	Adjacent	Fov030097	3
I7	45	Ovary	Cancer adjacent normal ovary tissue	-	-	Adjacent	Fov030429	3
I8	48	Ovary	Cancer adjacent normal ovary tissue	-	-	Adjacent	Fov031800	0
I9	53	Ovary	Cancer adjacent normal ovary tissue	-	-	Adjacent	Fov030486	1
I10	53	Ovary	Cancer adjacent normal ovary tissue	-	-	Adjacent	Fov032237	2
**J1**	**57**	**Ovary**	**Cancer adjacent normal ovary tissue**	**-**	**-**	**Adjacent**	**Fov030707**	**0**
J2	38	Ovary	Cancer adjacent normal ovary tissue	-	-	Adjacent	Fov030583	0
J3	53	Ovary	Cancer adjacent normal ovary tissue	-	-	Adjacent	Fov050407	0
**J4**	**59**	**Ovary**	**Cancer adjacent normal ovary tissue**	**-**	**-**	**Adjacent**	**Fov050391**	**1**
J5	48	Ovary	Cancer adjacent normal ovary tissue	-	-	Adjacent	Fov040519	1
J6	50	Ovary	Cancer adjacent normal ovary tissue	-	-	Adjacent	Fov040667	1
J7	52	Ovary	Cancer adjacent normal ovary tissue	-	-	Adjacent	Fov050436	1
J8	27	Ovary	Normal ovary tissue	-	-	Normal	Fov07N025	0
**J9**	**34**	**Ovary**	**Normal ovary tissue**	**-**	**-**	**Normal**	**Fov07N031**	**0**
**J10**	**19**	**Ovary**	**Normal ovary tissue**	**-**	**-**	**Normal**	**Fov07N013**	**1**

Supplementary Table 2. The immunofluorescence staining scores for CHTOP in various cell lines.

Biomarker	A2780	A2780-cis	IGROV1	IGROV1-cis	SKOV3	OV90	HOSE
CHTOP	2	3	1	3	3	3	0

Notes: "0" represents negative staining, and "1", "2" , "3" represent weak staining, medium staining, and strong staining, respectively.

Supplementary Table 3. The immunofluorescence staining results for CHTOP in cisplatin-resistant EOC cell lines

Biomarker	A2780-cis		IGROV1-cis	
	scr	KD	scr	KD
CHTOP	3	0	3	0

Notes: "0" represents negative staining, and "1", "2" , "3" represent weak staining, medium staining, and strong staining, respectively.

Supplementary Table 4. The immunofluorescence staining results for CHTOP in cisplatin-resistant EOC cell lines

Biomarker	A2780-cis		IGROV1-cis	
	**scr**	**KD**	**scr**	**KD**
CD44	3	3	3	3
CD105	3	3	3	3
ALDH1	3	3	3	3
Snail	3	0	3	1
C-Kit	2	0	3	0
Sox-2	3	3	3	3
Nanog	2	2	1	1
Oct-4	3	0	3	1

Notes: "0" represents negative staining, and "1", "2" , "3" represent weak staining, medium staining, and strong staining, respectively.
